# Repositioning metformin and propranolol for colorectal and triple negative breast cancers treatment

**DOI:** 10.1038/s41598-021-87525-z

**Published:** 2021-04-14

**Authors:** L. E. Anselmino, M. V. Baglioni, F. Malizia, N. Cesatti Laluce, C. Borini Etichetti, V. L. Martínez Marignac, V. Rozados, O. G. Scharovsky, J. Girardini, M. J. Rico, M. Menacho Márquez

**Affiliations:** 1 Instituto de Inmunología Clínica y Experimental de Rosario (IDICER, CONICET-UNR), Facultad de Ciencias Médicas (UNR), Santa Fe 3100, Rosario, Argentina; 2 CONICET, Rosario, Argentina; 3 Instituto de Genética Experimental, Facultad de Ciencias Médicas, Santa Fe 3100, Rosario, Argentina; 4 Instituto de Fisiología Experimental (IFISE, CONICET-UNR), Facultad de Ciencias Bioquímicas y Farmacéuticas (UNR), Suipacha 570, 2000 Rosario, Argentina; 5 CICYTTP IBIOGEM, CONICET, Diamante, Argentina; 6 Centro de Investigación y Producción de Reactivos Biológicos (CIPReB), Facultad de Ciencias Médicas, Suipacha 660, Rosario, Argentina

**Keywords:** Biochemistry, Cancer, Cell biology, Drug discovery, Molecular medicine, Oncology

## Abstract

Drug repositioning refers to new uses for existing drugs outside the scope of the original medical indications. This approach fastens the process of drug development allowing finding effective drugs with reduced side effects and lower costs. Colorectal cancer (CRC) is often diagnosed at advanced stages, when the probability of chemotherapy resistance is higher. Triple negative breast cancer (TNBC) is the most aggressive type of breast cancer, highly metastatic and difficult to treat. For both tumor types, available treatments are generally associated to severe side effects. In our work, we explored the effect of combining metformin and propranolol, two repositioned drugs, in both tumor types. We demonstrate that treatment affects viability, epithelial-mesenchymal transition and migratory potential of CRC cells as we described before for TNBC. We show that combined treatment affects different steps leading to metastasis in TNBC. Moreover, combined treatment is also effective preventing the development of 5-FU resistant CRC. Our data suggest that combination of metformin and propranolol could be useful as a putative adjuvant treatment for both TNBC and CRC and an alternative for chemo-resistant CRC, providing a low-cost alternative therapy without associated toxicity.

## Introduction

At present the number of deaths by cancer has not receded. Among the top 10 cancers with the highest mortality rates in the world, colorectal and breast cancers are in the second and fifth place, respectively^1^. This high mortality levels are also found in Argentina, where colorectal cancer is in second place and breast cancer in third place of deadly cancers^2^. Most cases of colorectal cancer (CRC) are diagnosed in advanced stages (III–IV), when the probability of development of local or distal recurrence and chemotherapy resistance is more elevated^3,4^. In breast cancer, the highest mortality rates occur in the triple negative type (TNBC), that represents 15–20% of all breast cancers cases^5^. This type of breast tumor is characterized by the lack of progesterone and estrogen receptors (PR and ER) combined with a low expression of the receptor 2 of human epidermal growth factor (HER2), which diminish the possibility of effective therapeutic resources.

Within the current treatment options for CRC a primary surgical resection of the tumor is often the first intervention, followed by radiotherapy and/or adjuvant chemotherapy. Since the 1950s, 5-fluorouracil (5-FU) has remained the mainstay of CRC chemotherapy^6,7^. In the recent years, other drugs have been developed and used in combination with 5-FU such as oxaliplatin, irinotecan and capecitabine^8^. The use of new monoclonal anti-EGFR (anti-epidermal growth factor receptor) or anti-VEGF (anti-vascular endothelial growth factor) antibodies such as Cetuximab and Bevacizumab has also allowed great advances in therapies^9^. However, almost half of CRC patients develop resistance to 5-FU based chemotherapies^10^, and a drug dose increase is not a good alternative due to the associated side effects (like hair loss, physical weakness, among others).

On the other hand, in TNBC treatment, the surgery combined with adjuvant and/or neoadjuvant chemotherapy is also the standard treatment for removable primary tumors. Within the available breast cancer chemotherapies, hormonal therapy (such as tamoxifen and aromatase inhibitors), and drugs targeted at HER2 (such as Trastuzumab) are not effective for TNBC subtype due to the lack of targets for these drugs^11^. Like in CRC, the standard treatment for TNBC is cytotoxic chemotherapy, which involves the administration of drugs that affect cell division, causing DNA damage, like fluorouracil, doxorubicin, or cyclophosphamide, alone or in combination with other drugs^11^. New treatment options for advanced TNBC are emerging, including poly-ADP-ribosyl polymerase (PARP) inhibitors, phosphoinositide 3-kinase (PI3K) pathway inhibitors, immune checkpoint inhibitors, and cyclin-dependent kinase (CDK) inhibitors; all these treatments have shown positive results in clinical trials in patients with TNBC^12,13^. However, these therapeutic options are not affordable for patients in low- and middle-income countries and some tumors develop resistance to these new treatments^14^ as tumor relapse rates for TNBC are high and metastatic events are very frequent^15^.

To counter this situation, new strategies are being implemented in cancer treatment, including improved early diagnosis (down-staging), discovery of reliable predictive biomarkers and development of novel adjuvant drugs/drug combinations^16^.

The development of a new drug is a process that can take 10–15 years. The number of drugs approved by the FDA (Food and Drug Administration) has been declining since 1995 and the investment in drug development has been gradually increasing, indicating that the cost of new drug development will continue to grow^17^. In this context, repurposing of drugs has emerged as an alternative to the classical pipeline of drug development. This methodology involves the exploration of existing drugs approved by FDA and other official regulatory agencies for new therapeutic purposes. Drug repositioning has two main advantages: the knowledge of the pharmacokinetics and toxicity of the drugs and their low costs and accessibility, since they are mostly generic^17^. This is also an approach of special importance for low- and middle-income countries, where conventional therapies are not economically accessible to a vast portion of the population^18,19^. Regarding CRC and TNBC several repositioning drugs are currently being tested in clinical trials. Furthermore, anticancer activity has been detected in vitro for a large number of drugs that are expected to be tested in preclinical trials^20–23^.

One of the most controversial drugs under reposition in recent years is metformin. This drug is already used in the so-called "metabolic therapies for cancer treatment"^24^. Metformin is a standard clinical drug used for type 2 diabetes mellitus (T2DM) and polycystic ovary syndrome treatment. Meta-analyses and epidemiological studies revealed that patients with T2DM have lower incidence of tumor development than healthy controls and that patients diagnosed with cancer have a lower risk of mortality when treated with metformin^25,26^. Many in vitro and in vivo experiments confirmed that metformin may inhibit proliferation of a variety of tumor cells, even though its anticancer mechanism has not been well characterized^27^. Recent studies demonstrated that antitumor effects of metformin may be associated to insulin and insulin-like growth factor levels decrease in peripheral blood that may lead to the inhibition of phosphoinositide 3-kinase/Akt/mechanistic target of rapamycin (mTOR) signaling or activation of AMP-activated protein kinase, which inhibits mTOR signaling^28^. In addition to its individual effect, it has been observed that metformin can act synergistically with other types of drugs; even the combination of metformin with traditional chemotherapy drugs has become a novel aspect of cancer therapy^29–31^.

Propranolol is a non-cardioselective β-adrenergic receptor blocker with reported antioxidant and anti-inflammatory properties mainly used for hypertension treatment, but also for angina pectoris, myocardial infarction, migraines, anxiety disorders and tremor^32^. Although its anticancer effects are not as well studied as those for metformin, retrospective analyses reported a decreased risk of head and neck, stomach, colon and prostate cancers in patients receiving propranolol^33^. The mechanism of action of the drug has not been elucidated yet; it seems to act differentially depending on the type of cancer. It has been reported that propranolol could be acting in a β-adrenoreceptor independent manner through the HIF-1α-VEGF-A angiogenesis axis, with effects mediated through the PI3K/Akt and p38/MAPK pathways^34^. Propranolol is also an inhibitor of the PAP (phosphatidate phosphatase) activity of lipins, which probably explains why it inhibits autophagy flux^35,36^. However, far from its effect in the hypertension treatment, it seems that the strongest action of propranolol in cancer is related to alterations in cell metabolism, just like metformin^37,38^.

We previously reported a clear effect of metformin and propranolol combination (M + P) on TNBC cell models, affecting proliferation, mitochondrial activity and invasion capacity of the cells. M + P combination was effective on immunocompetent mice models of the same type of cancer, reducing tumor growth and preventing metastasis development with no symptoms of toxicity^23^. To go deeper with these findings, in this research we explored the steps leading to lung metastasis formation in TNBC and developed in vivo adjuvant models demonstrating that M + P combination could be proposed as a good adjuvant therapy after tumor resection. Besides, we characterized the effect of M + P in CRC models, both in vitro and in vivo, evaluating the impact of M + P treatment on cellular processes such as migratory capacity, apoptosis and proliferation, for which the combination also proved to have a potential antitumor effect.

## Results

### Sensitivity of CRC cells to repurposing drugs: combination of Metformin and Propranolol affects CRC cells viability

**Figure 1:**
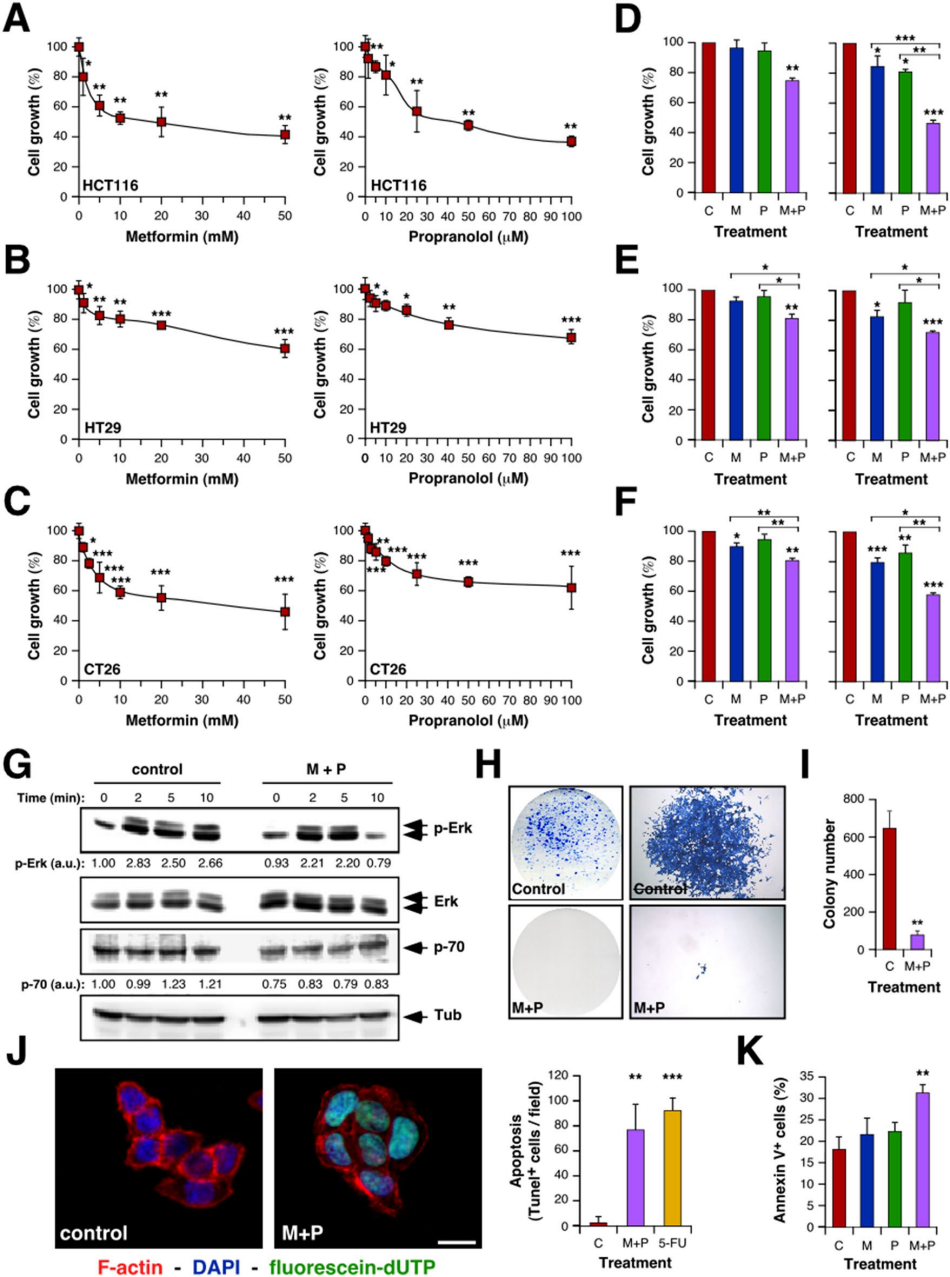
Characterization of the effect of Met and Prop on CRC cells viability. CRC cells were cultured in the presence of the indicated doses of Met or Prop (**A**–**C**) during 36 h. The number of living cells was estimated by tetrazolium salts reduction method (n = 3). (**D**–**F**) HCT116 (**D**), HT29 (**E**) and CT26 (**F**) cells were treated for 24 h with Met (M; 1 mM left, 2.5 mM right), Prop (P; 1 µM left, 2.5 µM right) or a combination of them (M + P) and living cells were estimated as before (n = 3). (**G**) Western blot analysis showing the phosphorylation and expression status of Erk and p70 in serum-stimulated HCT116 cells. Cells were treated for 30 min with M + P (M 2.5 mM; P 2.5 µM) before released in complete media. Tubulin was used as loading control (n = 3). Quantification of phosphorylation levels is indicated. (**H**,**I**) Cells (500 cells/well) were cultured in the presence of M + P during 8 days. Colonies were stained (**H**) in order to allow quantification (**I**). (**J**) TUNEL assay (left, scale bar = 100 µm) and quantification (right) of HCT116 apoptotic cells after M + P treatment. 5-FU was used as a control (n = 3). (**K**) Quantification of apoptotic (annexin V^+^) HCT116 cells by flow cytometry after single or combined treatment (n = 3).

To evaluate the potential use of drugs under reposition for CRC treatment, we explored the effect of 10 selected drugs with impact on different cellular metabolic pathways (listed in Table SI) on the proliferation of two different human CRC cell lines, HCT116 and HT29. All the drugs tested were able to reduce cell growth in a dose-dependent manner (Fig. 1A,B; Figure S1A; Table SI). We have previously described the benefits of M + P combination to treat TNBC^23^. Since both, Met and Prop, showed a clear effect on human CRC cell lines, we extended this observation to a mouse CRC cell line (CT26; Fig. 1C) and then explored the effect of M + P combination on all the CRC cell lines under study (Fig. 1D–F). By combining M + P cell proliferation was significantly reduced even at doses as low as 1 mM for Met and 1 μM for Prop, and in all cases, combination of M + P was more effective than any individual treatment. Accordingly, cells pre-treated with M + P showed a reduced signaling response to proliferative stimulus, observed by a decrease level of Erk phosphorylation and reduced expression of p70 ribosomal protein S6 kinase after serum activation (Fig. 1G; Figure S1B). A failure in reaching normal Erk phosphorylation levels after serum-stimulus was also observed in a TNBC cell model (Figure S1C).

To further extend these observations, we analyzed the effect of M + P combination on the clonogenic capacity of CRC cells. This combination was able to reduce the ability of single cells to grow into colonies (Fig. 1H,I; Figure S1E,F). As expected from our proliferation analysis, not only the number of colonies was affected, but also the size of the colonies under treatment was significantly reduced (Fig. 1H,I; Figure S1D–F).

Also, as it was the case for TNBC, M + P combination was able to promote CRC cell death by apoptosis, as observed by Terminal deoxynucleotidyl transferase dUTP nick end labeling (TUNEL) assay and Annexin V staining (Fig. 1J,K).

### Metformin and Propranolol combination has a clear impact on metastasis-related events

**Figure 2:**
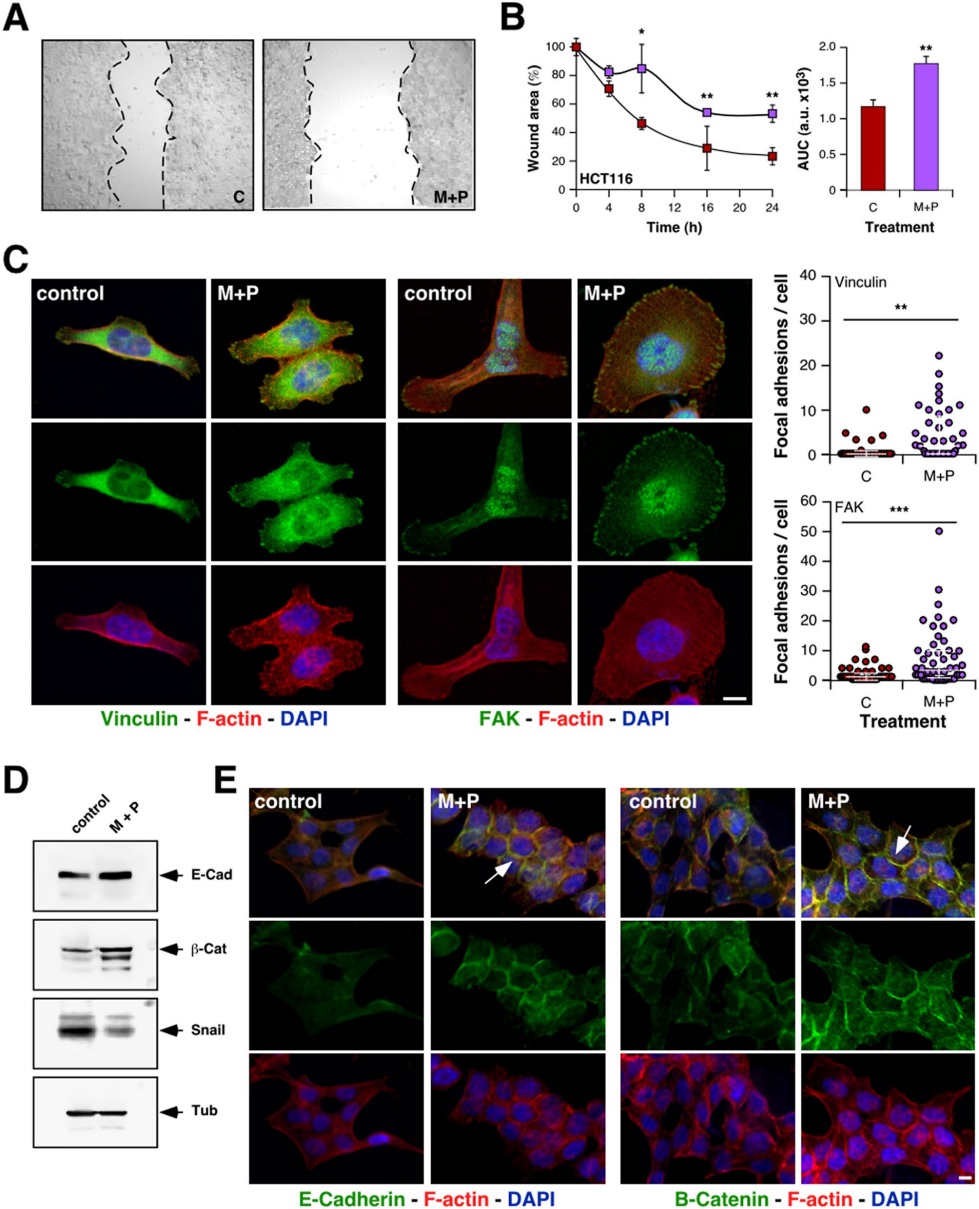
Combination of M + P affects EMT in CRC cells. (**A**,**B**) The migratory ability of HCT116 cells was estimated by measuring closure of the initial wound. Pictures were taken (**A**) to allow quantification of healing (**B**) by using the Image J software (AUC, area under the curve). (**C**) Focal adhesions were highlighted by Immunofluorescent detection of vinculin (green color, left panel) and FAK (green color, middle panel) in HCT116 cells counterstained with F-actin and nuclei (red and blue colors respectively, scale bar = 10 µm) and quantified (right panel). (**D**) Representative immunoblots showing modulation of EMT markers expression by M + P treatment. For loading control, we used the abundance of endogenous tubulin (n = 3 independent experiments). (**E**) Representative immunofluorescence analysis showing the abundance and subcellular localization of endogenous E-cadherin (green color, left panel), β-catenin (green color, right panel), rhodamine phalloidin-stained F-actin (red color), and DAPI-stained nuclei (blue color). Arrows indicate increased detection at cellular junctions (scale bar = 10 µm).

To continue exploring the putative benefits of CRC treatment with the combination of drugs under study, we characterized the effect of M + P on cell migration by classical wound-healing assays. Although individual treatments were able to partially inhibit HCT116 cells migration (Figure S2A), combined treatment showed a stronger inhibition of the migratory capacity of all the CRC cell lines analyzed (Fig. 2A,B; Figure S2B–D).

Focal adhesions are dynamic structures that form mechanical links between cytoskeletal intracellular actin bundles and the extracellular substrate. Under normal conditions, focal adhesions are quite stable, but in motile cells they should be constantly assembled and disassembled. Dynamic assembly and disassembly of focal adhesions plays a central role in cell migration. Stimulation of focal adhesions formation increases the attachment of cells to substrates, leading to cells with spread morphology. In contrast, directional migration of the cell requires continuous formation and turnover of focal adhesions at the leading edge of the cell body and release of this attachment at the rear^39^. To deeply explore the effect of M + P combination on CRC cells migratory behavior, we analyzed the impact of this treatment on the number and distribution of focal adhesions, and actin cytoskeleton organization. The observation of M + P-treated CRC cells under the microscope revealed a more widespread morphology than control cells (Fig. 2C), with an increased number of focal adhesions per cell as evidenced by vinculin and FAK (focal adhesion kinase) immunostaining (Fig. 2C). Not only the number of focal adhesions was increased, but also, they were wider and distributed all over the cell body (Figure S2E,F), suggesting a stronger substrate attachment and a less migratory morphology. According to this observation, actin staining revealed a reduction of filopodia and ruffling lamellipodia in treated cells, and by all accounts more stabilized microtubule cytoskeleton (Figure S2G).

### Combination of Metformin and Propranolol promotes the acquisition of epithelial traits on CRC cells in vitro

Aggressive tumor cells gain an invasive and metastatic phenotype trough a mechanism that recapitulates several aspects of epithelial to mesenchymal transition (EMT). To extend our observations, we explored the putative effect of M + P treatment on the expression of epithelial markers. Both by Western blot and immunofluorescence (Fig. 2D,E; Figure S2H) we detected a clear increase in the expression of E-cadherin and β-catenin after 24 h incubation with M + P. Concomitantly with the increased expression of these epithelial markers, we observed reduced levels of the transcription repressor Snail. Indeed, localization of these epithelial proteins in the cell membrane was increased in treated-cells, most likely as part of adherens junctions (Fig. 2E), suggesting that this treatment could prevent EMT in CRC. Nevertheless, this effect of M + P on EMT seems to be CRC-specific as no change in E-cadherin expression was observed after treatment in a TNBC model (Figure S2I).

### Metformin and Propranolol combination prevents development and progression of colorectal tumors

**Figure 3:**
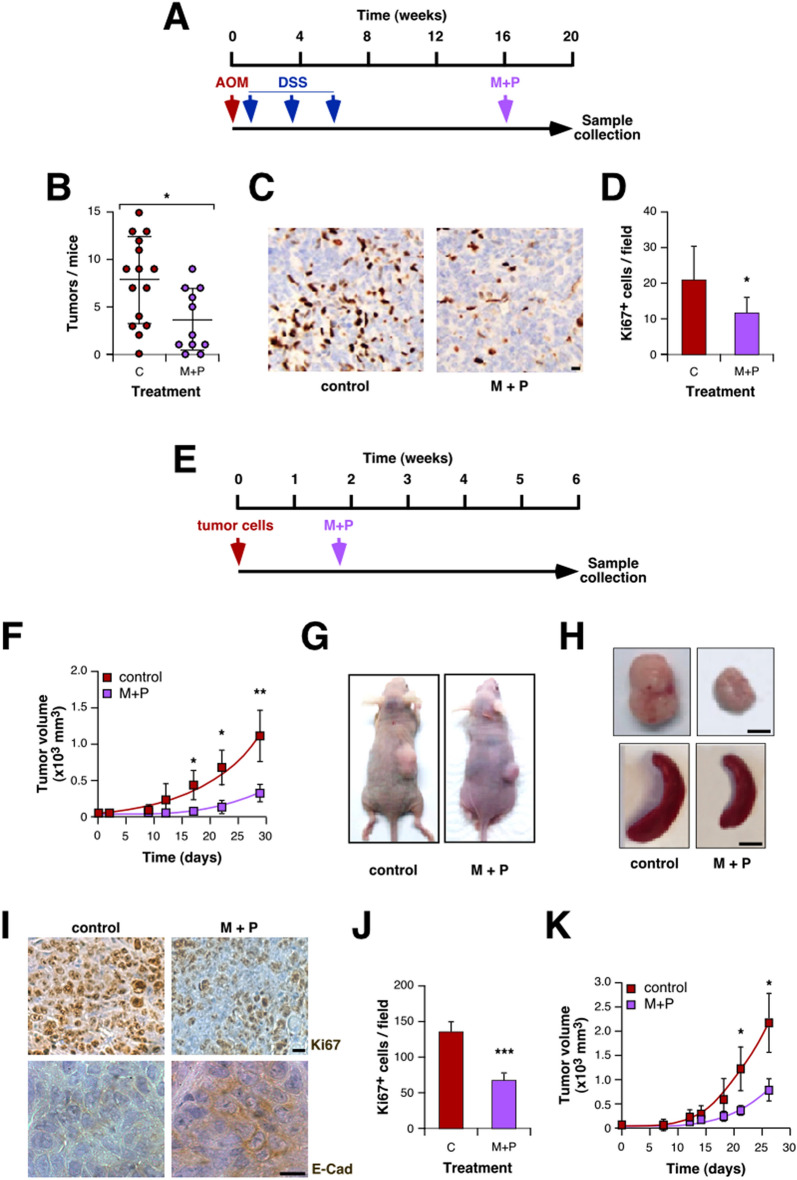
Combination of Met and Prop prevents development and growth of CRC. (**A**) Scheme of the chemical-induced carcinogenic model (AOM, azoxymethane; SDS sodium dextran sulfate). (**B**) After carcinogenesis, mice intestines were collected and tumors visualized under a stereoscopic microscope and quantified. (**C**,**D**) Proliferation of tumor cells was estimated by Ki67 immunostaining (**C**) and quantified (**D**). (**E**) Scheme of the xenographic model used in this section: immunodeficient mice were subcutaneously challenged with HCT116 cells. Ten days later treatment was added to the drinking water. (**F**–**J**) The tumor size was measured biweekly with a caliper and volume estimated (**F**, time = 0 indicates the beginning of M + P treatment). At the end of the experiment, animals were sacrificed for necropsy (**G**,**H**). (**I**) Representative pictures of tumors immunostained for Ki67 and E-cadherin (scale bar = 100 µm). (**J**) Tumor cells proliferation was estimated by quantification of Ki67-positive cells. (**K**) Immunocompetent mice were subcutaneously challenged with CT26 cells following the diagram shown in (**E**). Tumor volume was estimated by measuring diameters biweekly with a caliper.

To validate our in vitro data, we performed two different in vivo approaches. For all the in vivo experiments we choose Met and Prop doses previously described to treat diabetes or hypertension on mouse models of these diseases, with no toxicity associated^40,41^. First, we evaluated the effect of M + P treatment on the evolution of chemically induced intestinal tumors (Fig. 3A). Through the visualization of samples under a stereoscopic microscope (Figure S3A) the number of tumors was counted revealing that M + P treatment reduced significantly tumor incidence (Fig. 3B). There were no clear histological differences between tumors from treated or control animals (Figure S3B), but tumor cell proliferation was significantly affected by treatment as observed by Ki67 immunostaining (Fig. 3C,D).

To complement this result, we analyzed the putative benefits of M + P administration on xenograft models (Fig. 3E). Animals injected with HCT116 cells treated with M + P showed a reduced tumor growth rate compared to untreated mice (Fig. 3F,G). Accordingly, tumor volume at the end of the experiment was smaller for treated animals (Fig. 3H) and there was no evidence of splenomegaly or liver metastasis for that group (Fig. 3H, Table SII). Histological analysis of samples collected at the end of the experiment showed no clear morphological difference between groups, and the absence of metastatic growth in lungs derived from M + P-treated mice (Figure S3C,D, Table SII). To further confirm if the phenotypes observed in vitro were reproduced in vivo, we performed immunohistochemistry to evaluate proliferation and EMT, corroborating that tumors derived from M + P-treated animals showed a reduced proliferative index as observed by Ki67 immunostaining (Fig. 3I,J) and increased expression of E-cadherin (Fig. 3I). A similar CT26-based allograft model on immunocompetent BALB/c mice allowed us to confirm the effect of M + P treatment on tumor growth. Once again, treatment reduced CRC tumor progression (Fig. 3K) and reduced lung metastasis development with no clear histological difference between groups (Figure S3E–H, Tables SIII and SIV). Noteworthy, there were no signs of toxicity associated with treatments as seen by analysis of mice weight evolution (Figure S3I–K) and general behavior (data not shown).

### Metformin plus Propranolol combined treatment affects different steps leading to metastasis development

**Figure 4:**
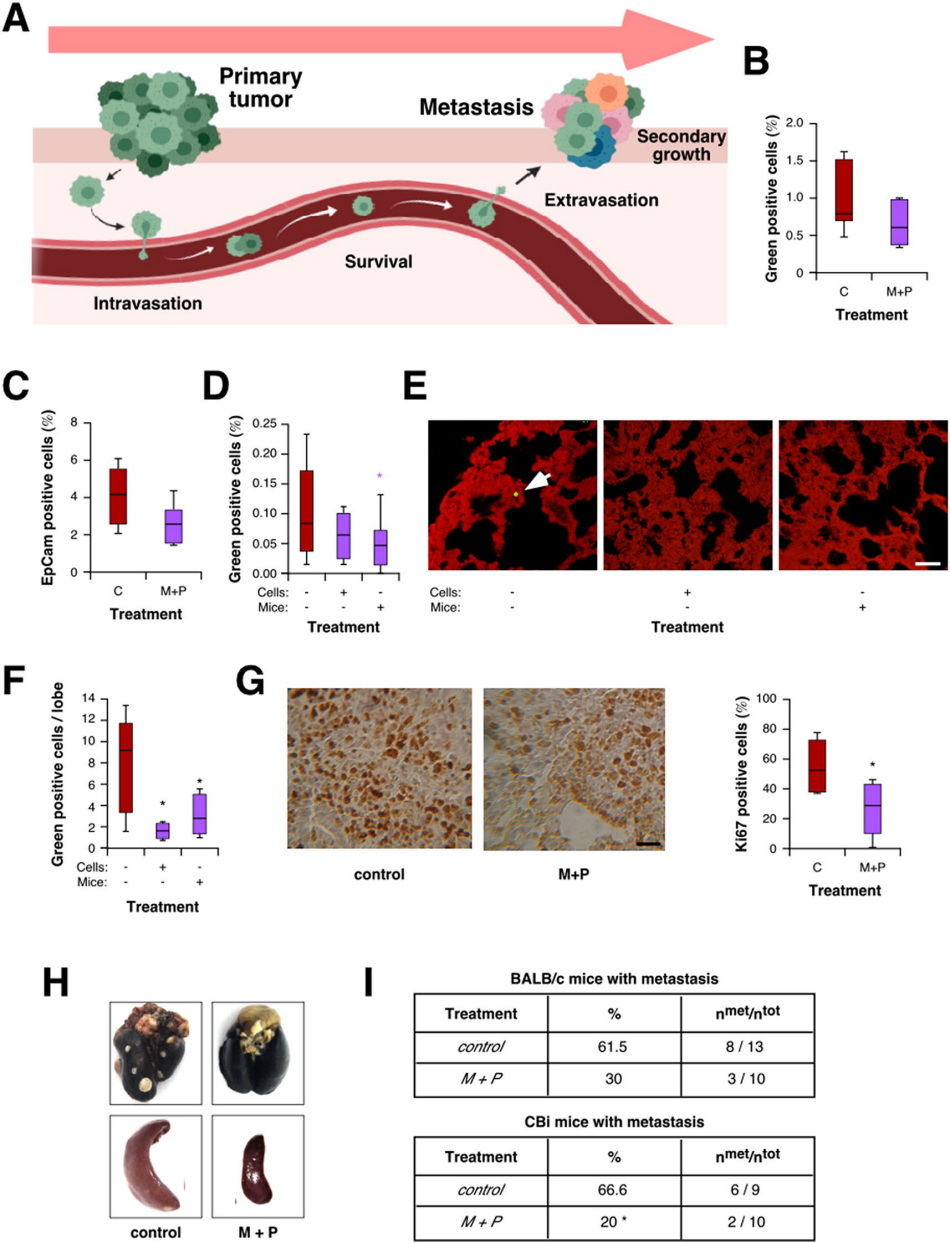
M + P combination prevents secondary tumor growth by affecting different steps leading to metastasis. (**A**) Scheme of the different steps involved in metastasis development analyzed in this section. (**B**,**C**) Intravasation was estimated by flow cytometry quantification of green-positive cells in the bloodstream after orthotopical injection of 4T1-GFP cells (**B**) and flow cytometry quantification of EpCam^+^CD45^−^CD34^−^ cells present in blood after orthotopical injection of 4T1 cells (**C**; p = 0.066). (**D**) Cell tracker stained 4T1 cells were i.v. injected and survival was estimated 48 h later by flow cytometry as described in Material and Methods. (**E**,**F**) Representative pictures (**E**, scale bar = 100 µm) and quantification (**F**) of green positive cells in the lung parenchyma. (**G**) Proliferation of cells in secondary nodes was evaluated and quantified by Ki67 immunostaining of lung metastasis (scale bar = 100 µm). (**H**,**I**) BALB/c and CBi mice were orthotopically injected with 4T1 (**H** and **I**, top panel) or M-406 (**I**, bottom panel) cells, respectively. Tumors were surgically removed. M + P was added to the drinking water of treated animals. At the end of the experiment lungs and spleen were collected (**H**) and observed for the presence of metastasis (**I**).

Our data indicate an effect of M + P treatment on metastasis development both in CRC and TNBC models. To further characterize in vivo the processes leading to metastasis development affected by this treatment (Fig. 4A), we used 4T1 TNBC cells, which are able to colonize lungs at high frequency. To begin with this characterization, we evaluated the effect of M + P on intravasation by two different approaches. First, we injected orthotopically 4T1 cells expressing GFP in BALB/c mice and analyzed the presence of green-positive cells in the bloodstream by flow cytometry (Fig. 4B). In a parallel assay, we quantified the presence of cells in blood expressing the epithelial marker EpCam (Fig. 4C). Interestingly, both approaches allowed us to observe a decrease, although not significant, in the percentage of intravasating cells, suggesting that M + P treatment could affect, at least partially, the process of intravasation.

Then, we evaluated the effect of M + P on the ability of cancer cells to survive in the bloodstream. To address this, we injected intravenously green-labelled 4T1 cells in mice under treatment and evaluated the presence of green cells by flow cytometry, noting that administration of M + P significantly diminished survival of circulating tumor cells in the blood flow (Fig. 4D). To continue with this characterization, we assessed the effect of M + P on the ability of cells to successfully complete lung extravasation by analyzing the presence of green-positive cells in lung parenchyma by confocal microscopy 48 h after injection. Through this approach, we detected a significant decrease in the number of tumor cells present in the lungs of treated-mice or mice injected with pre-treated cells (Fig. 4E,F). Interestingly, pre-treatment of cells with M + P affected partially survival and significantly lung extravasation (Fig. 4D–F).

Finally, we checked the potential of cells to growth in a secondary niche by Ki67 immunostaining of 4T1 metastatic nodes, observing that M + P treatment decreased significantly the ability of cells to proliferate in the lungs (Fig. 4G).

### Combination of Metformin and Propranolol could be effective as adjuvant therapy

Once observed the effect of M + P on different steps leading to metastasis, we set up a model to evaluate the putative use of this drug combination as adjuvant therapy after surgical removal of primary tumors. As breast cancer cells are easy to inject orthotopically, and tumor surgical extirpation is simple to perform, we decided to use 4T1 cells for this approach. After surgery, animals were randomly distributed in two groups, one of them receiving M + P in the drinking water. Upon sacrifice, we observed a reduction, although not significant, in the number of animals with lung metastasis and splenomegaly (Fig. 4H,I). Interestingly, when tested on a different TNBC model (based on the mammary adenocarcinoma M-406^42^), we obtained a significant reduction in the incidence of animals with lung metastasis (Fig. 4I), suggesting that M + P treatment could be useful as adjuvant treatment to prevent metastasis development. Once again, no sign of toxicity was associated to the treatment (data not shown).

### M + P treatment is effective on 5-FU resistant CRC cells

**Figure 5:**
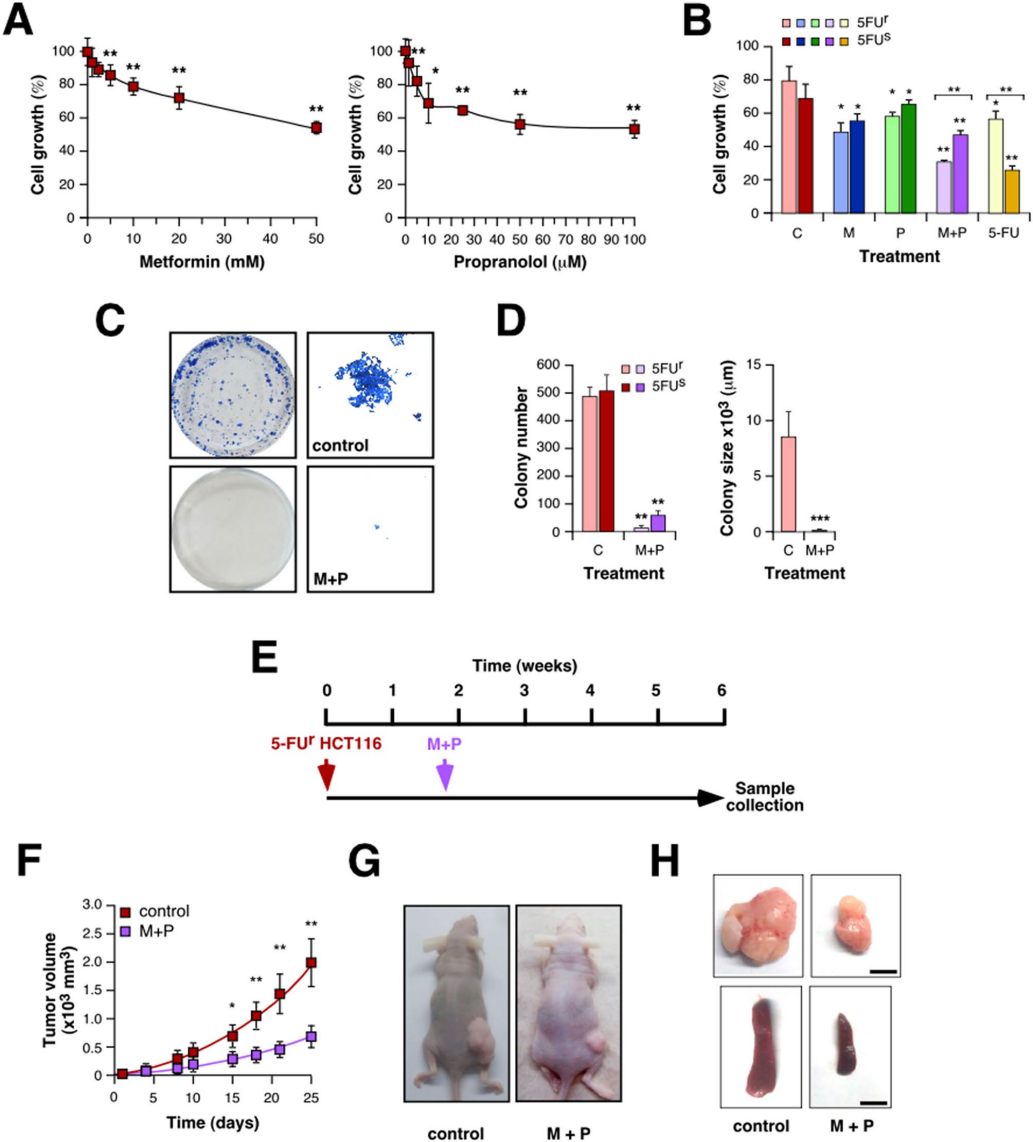
Combination of M + P prevents growth of 5-FU-resistant CRC cells. (**A**) 5-FU resistant HCT116 cells were cultured in the presence of the indicated doses of Met (left) or Prop (right) during 36 h. The number of living cells was estimated by tetrazolium salts reduction method (n = 3). (**B**) 5-FU sensitive (5FU^s^) and resistant (5FU^r^) HCT116 cells were cultured in the presence of Met (M, 2.5 mM) Prop (P, 2.5 µM), a combination of both or 5-FU (5 µM) as described before. (**C**,**D**). 500 cells were cultured in the presence of M + P during 8 days. Colonies were stained (**C**) in order to allow quantification (**D**). Colony size for 5-FU resistant cells was estimated (**D**, right panel). (**E**) Scheme of the xenographic model used in this section. (**F**) Immunodeficient mice were subcutaneously challenged with 5-FU resistant HCT116 cells. Ten days later, treatment was added to the drinking water. The tumor size was measured biweekly with a caliper and volume estimated (time = 0 indicates the beginning of M + P treatment). At the end of the experiment, animals were sacrificed for necropsy (**G**,**H**).

Once evaluated the effect of combining M + P on CRC and TNBC and confirmed the effect of this treatment preventing metastasis development, we wondered if this drug combination could be also effective in colorectal tumors resistant to conventional therapies. As it was described, 5-FU is one of the most prescribed drugs to treat CRC, but many tumors develop resistance to this therapy. In order to evaluate if M + P treatment could be effective also against 5-FU-resistant tumors, we tested the effect of this combination on 5-FU resistant HCT116 cells. Noteworthy, these resistant cells are sensitive to individual treatments (Fig. 5A) but particularly sensitive to M + P combination (Fig. 5B). Interestingly, the effect of M + P on proliferation was more pronounced on 5-FU resistant cells when they were compared to parental cells, although there was no difference in the effect of individual treatments under the same conditions (Fig. 5B). Accordingly, M + P treatment also affected clonogenicity of 5-FU-resistant cells, causing a decrease in colonies size (Fig. 5C,D). Interestingly, pre-treatment with M + P was sufficient to confer 5-FU sensitivity to 5-FU resistant HCT116 cells (Figure S4A).

In order to corroborate these in vitro data, we performed in vivo studies by injecting 5-FU-resistant HCT116 cells subcutaneously in nude mice (Fig. 5E). Animals treated with M + P showed a reduced tumor growth rate compared with untreated mice (Fig. 5F,G), a concomitant decrease in tumors size at the end of the experiment and no signs of splenomegaly (Fig. 5H). As before, histological analysis of tumors showed no clear differences among groups (Figure S4B) but a reduced number of lung metastasis for treated animals visualized under the microscope (Figure S4C, Table SV). As expected, no sign of toxicity was associated to the treatment regarding mice weight and general behaviour (Figure S4D and data not shown).

## Discussion

Cancer continues to be one of the main causes of death with a high global prevalence despite the numerous advances made in the last decade. CRC is expected to record the highest mortality among digestive cancers, as its development is influenced by age, dietary habits, smoking, alcohol consumption, genetic predisposition, obesity, diabetes, and sedentary lifestyle^43^. On the other hand, TNBC represents a highly heterogeneous subtype of breast cancer with a poor prognosis, high rates of recurrence and metastasis. For both CRC and TNBC, chemotherapy (after surgery when possible) is the primary established systemic treatment for patients in early and advanced stages of the disease^4,44^. While these treatment regimens are efficient and improve overall survival, serious side effects such as nausea and vomiting, anemia, and the risk of infection caused by immunodepression, affect the patient’s quality of life. Therefore, there is an urgent need to develop new clinically effective and well-tolerated therapeutic approaches.

Drug repositioning or repurposing refers to the establishment of new medical indications for already known drugs, including approved, discontinued, archived, and experimental drugs^45^, that has risen as an interesting alternative strategy to de novo drug synthesis. Repurposing existing approved medicines offers a cost-efficient way to increase treatment options for oncologic patients, especially for tumors showing relentlessly rising incidence rates and an unmet success of new anticancer agents^46^.

Previously, we reported that combination of M + P was effective preventing TNBC growth and metastasis development^23,47^. In this study, we explored the effect of this combination of drugs on CRC and dissected the steps involved in metastasis spreading affected by these drugs.

To begin our study, we explored the effect of different drugs under reposition on CRC cell lines. As suggested by previous reports^20,48^, we observed that all the drugs tested affected CRC cell proliferation in a dose-dependent manner. As we described formerly that Met and Prop can act synergically on TNBC cells, it was tempting to explore the action of their combination on CRC cells. Thus, we clearly observed that M + P had a negative effect on CRC cell growth, even at smaller doses than the ones used previously for TNBC^23^. Although the antiproliferative effect of Met on CRC cells was widely reported^49–55^, there are scarce reports aiming to characterize the antitumor effect of Prop on this cancer type^56,57^. For example, it was previously reported that Met promoted growth inhibition and apoptosis by activation of the AMPK-mTOR pathway in human colorectal cancer cells^58^. Also, the clonogenic capacity of CRC tumor cells was also affected by our treatment in all three in vitro models explored, reducing both colonies number and size. In agreement with our observation, it was recently described that Met transiently inhibits colorectal cancer cell proliferation and clonogenicity through AMPK activation and increase ROS production, which suppressed the mTOR pathway and its downstream targets S6 and 4EBP1^59^.

Interestingly, we observed antiproliferative effects of Prop at lower doses than the ones described previously. Moreover, the combination of drugs allowed decreasing Met and Prop doses to 1 mM and 1 μM respectively, without losing their effect on viability. These concentrations can be considered as clinically relevant as they are comparable to the ones found in plasma of patients receiving Met or Prop^60–62^, which allow us to speculate with future translation to phase I studies or compassionate use for this combination of drugs.

The reported effect on proliferation correlated with reduced levels of Erk phosphorylation and expression of p70 ribosomal protein S6 kinase observed after serum stimuli when cells were pre-treated with M + P. Defective Erk phosphorylation in the presence of M + P was also observed after proliferative induction in 4T1 cells, suggesting that the underlying mechanism impinging tumor cell growth of this combination of drugs is maintained independently of the cell type.

Interestingly, the impact of M + P on proliferation is conserved in vivo, as we observed that combined treatment was effective reducing significantly the growth of HCT116 and CT26 tumors in mice, with no associated symptoms of toxicity. Although there is not direct correlation between Met dosage in pre-clinical models and those used in clinical trials, plasma Met concentration in treated-mice is reported to be close to that observed in diabetic patients^62,63^. Ongoing Met dosage in clinical trials for cancer treatment ranges from 500–1700 mg/day^64^. Regarding Prop, dose varies by indication from 160–320 mg/day for hypertension to 120–240 mg/day for angina. Interestingly, Prop dose used to treat infantile hemangioma can be raised to 3 mg/kg/day^61^, close to the one used in our pre-clinical models.

Through in vitro assays we observed that M + P combination not only affected CRC cells growth, but also compromised their clonogenic capacity and it was capable to promote apoptosis. The ability of Met to hit HCT15 CRC cell colony formation was previously reported in combination with the dual PI3K/mTOR inhibitor Dactolisib^65^. As it was described that Prop inhibits PI3K activity in hemangioma^66^, we speculate that the clear effect of M + P on clonogenicity may be related to the enhanced suppression of PI3K/AKT/mTOR pathway, hypothesis that should be addressed with additional work. Regarding CRC programmed cell death, there are several reports for Met and fewer for Prop demonstrating that these drugs are able to induce apoptosis in different in vitro CRC models^51,56,67,68^. We demonstrated in this study that M + P combination is able to elicit CRC cells apoptosis at lower doses than the ones formerly reported, perhaps associated to an increase of the cell cycle arrest at G0/G1.

The decreased capacity of CRC cells to form colonies and the increased apoptosis in the presence of M + P could be partially responsible for the reduced tumor burden developed by mice after chemically induced carcinogenesis.

As metastasis is the major cause of death in CRC patients, we explored the effect of M + P on different metastasis-related cellular events. As expected from previous reports by other groups^57,69,70^ both Met and Prop affected CRC cells migration in vitro. Noteworthy, combined treatment caused significantly stronger migration impairment for all the cell types analyzed. Not only M + P affected cell migration, but also modulated CRC cells morphology, cytoskeleton arrangement, focal adhesion number and distribution, and maintenance of epithelial features. Regulation of these cell events closely related to metastasis development could account for the decrease in secondary tumor growth observed in our CRC in vivo models. Indeed, in vivo dissection of the steps leading to metastasis demonstrated that M + P combination could reduce their development affecting EMT, intravasation and cancer cell survival in the blood stream, extravasation, and the ability to proliferate in a secondary niche. Moreover, our results provide evidence on the potential of M + P treatment as adjuvant therapy after primary tumor resection based on 4T1 and M-406 TNBC models. Although our results are based in two different TNBC models, it is important to note that TNBC is a heterogeneous disease lacking of targeted therapeutics and current treatments lead to heterogeneous patient outcomes.

Unfortunately, part of the low survival rates of CRC patients are associated to drug-resistance, as nearly half of the patients with metastatic CRC are resistant to 5-FU based therapy^10^. Noteworthy, we observed that M + P combination proved efficacy against metastatic dissemination and proliferation of 5-FU resistant CRC, suggesting that target pathways of M + P treatment are not modified by the genetic/epigenetic changes involved in 5-FU resistance. As described before for Met^71–73^, we also noted that combined treatment sensitized resistant cells to 5-FU treatment.

Altogether, our data allow us to suggest M + P combination as another possibility of treatment for chemo-resistant CRC and as a putative adjuvant treatment both for CRC and TNBC, avoiding the toxicity generally associated to conventional chemotherapies and providing a low-cost treatment that could be beneficial in particular for low and middle-income countries.

## Materials and methods

### Cell culture

HCT116 and HT29 human colon adenocarcinoma cell lines (kindly provided by Dr. Martínez Marignac from UADER- collection) were cultured in DMEM and supplemented with 10% fetal bovine serum (FBS; Natocor, Argentina), penicillin (10 μg/ml), streptomycin (100 μg/ml) and l-glutamine (2 mM). 4T1 (mammary) and CT26 (colon) BALB/c mice-derived carcinoma cells were cultured in RMPI medium supplemented with 10% FBS, penicillin (10 μg/ml) and streptomycin (100 μg/ml). Cells were incubated at 37 °C with 5% CO_2_. 4T1 cells were kindly provided by Dr. N. Zwirner (IBYME-CONICET). CT26 cells were kindly provided by Dr. V. Segatori (Universidad Nacional de Quilmes). Identity of cells was confirmed by standard STR analysis. Cells were tested periodically for Mycoplasma.

### Proliferation assays

For in vitro cell viability assays 5 × 10^3^ cells were seeded on 96-well plates and incubated for 36 h at 37 °C at a 5% CO_2_ atmosphere with increasing doses of the indicated drugs to reposition and some combinations of them (Table SVI). At the end of the incubation time, treatments were removed and an MTT assay (Sigma Aldrich) was performed following manufacturer indications. Subsequently, cells were lysed with DMSO and the amount of metabolized MTT was measured by spectrophotometry reading absorbance at 570 nm. Viability was expressed as the percentage of control untreated samples. The concentration of drugs that decreased cell proliferation by 50% (IC50) as compared to controls was calculated with GraphPad Prism 7 (GraphPad Software, La Jolla, CA, USA).

### Apoptosis

A TUNEL assay was performed to evaluate the apoptotic effect of the M + P combination on HCT116 cells. Briefly, 5 × 10^3^ HCT116 cells/coverslip were seeded, one group was treated for 16 h with 2.5 mM M plus 2.5 µM P, a second group was designated as negative control without treatment and a third group was designated as positive control treated with 5-FU 5 μM. The test was performed using In Situ Cell Death Detection Kit (Roche) according to the provider's protocol. TUNEL positive cells were quantified with a standard immunofluorescence microscope.

In parallel, apoptosis was also examined by flow cytometry. Cells were treated with 2.5 mM of M, 2.5 μM of P, or the combination, for 16 h, then collected, washed and stained with Annexin V-FITC (AP-Biotech) and propidium iodide (Sigma). Cells were analyzed using a FACSAria II flow cytometer (BD Biosciences) and apoptosis was estimated using the FlowJo software.

### Cell migration assay

HCT116 and HT29 cells were plated and cultured until reach subconfluency. A wound was made on the cell monolayer with the help of a yellow tip. To analyze cellular motility in non-proliferative conditions, cells were put on media supplemented with 0.1% FBS in absence or presence of treatments (2.5 mM M; 2.5 μM P) and maintained under these conditions during the whole assay. Cellular motility was estimated by measuring the closure of the initial wound. Photos were taken at 4, 8, 16 and 24 h using a Nikon Livecell microscope. Quantification of healing was performed using the Image J software. Areas under the curve were determined as described before^23^.

### Cells immunostaining techniques

5 × 10^3^ HCT116 cells were cultured in coverslips for 24 h, treated for the indicated time, and then fixed in 4% paraformaldehyde and permeabilized with a 0.3% PBS-Triton solution for 10 min. 2% equine serum (Gibco) solution was used to block for one hour at room temperature, followed by incubation with a primary antibody under the same conditions. The antibodies used were: α-Tubulin (Sigma, 1:1000 dilution), Vinculin (Santa Cruz Biotechnology, 1:50 dilution), FAK (Santa Cruz Biotechnology, 1:50 dilution), β-catenin (Santa Cruz Biotechnology, 1:100 dilution), and E-cadherin (Santa Cruz Biotechnology, 1:50 dilution). A second incubation of one hour in the dark with an appropriate Alexa Fluor 488-conjugated secondary antibody (Invitrogen, 1:500 dilution) was performed to allow protein detection. Between each step coverslips were washed three times with PBS for 5 min. Complementary staining with DAPI (Sigma, 1:10,000 dilution) and phalloidin-rhodamine conjugate (Invitrogen, 1:2000 dilution) was carried out to observe cell nucleus and actin skeleton, respectively. Finally, coverslips were fixed to a slide with 4 μl of Mowiol and observed under a Nikon C2 confocal microscope. Distribution and size of focal adhesions between groups was performed with the ImageJ software. Angle in degrees for each focal adhesion relative to the center of the nucleus of each cell was measured. Data were entered into the GraphPad Prism v7 software where a frequency histogram was constructed.

### Immunoblotting

Cells were plated on 6-well dishes until reach 70% of confluence and then treated for the indicated times with M + P. Cells were lysed in RIPA buffer supplemented with protease inhibitor cocktail (Roche Diagnostics, Mannheim, Germany) and scraped off the dish to perform protein extracts. Protein concentration was determined using the Lowry assay and gels were loaded with 40 µg protein after denaturation for 5 min at 95 °C in SDS-PAGE protein sample buffer. Samples were separated on 8% SDS–polyacrylamide gels and transfer to PVDF membrane (Amersham Hybond P, GE Healthcare Life Sciences) was carried out for 90 min with constant current at 4 °C. Membranes were blocked with 5% (w/v) skim milk in TBS-Tween (50 mM Tris, 150 mM NaCl, 0.05% Tween, pH 7.5) for 60 min at room temperature and further incubated with the indicated primary antibody overnight at 4 °C. Antibodies used included α-Tubulin (Sigma, 1:1000 dilution), total Erk1/2 (Santa Cruz Biotechnology, 1:200 dilution), β-catenin (Santa Cruz Biotechnology, 1:1000 dilution), phospho-Erk1/2 (residues Thr202/Tyr204; Cell Signaling, 1:1000 dilution), E-cadherin (Santa Cruz Biotechnology, 1:500 dilution), p70 S6 kinase α (Cell Signaling, 1:1000 dilution) and SNAIL/C15D3 (Cell Signaling, 1:1000 dilution). After three washes with TBS-Tween to remove the primary antibody, membranes were incubated with the appropriate peroxidase-conjugated secondary antibody (Biorad, 1:5000 dilution) for one hour at room temperature. Detection was done by chemiluminescence (Bio-Lumina; Kalium Technologies, Argentina) using a Licor C-Digit Blot Scanner (LI-COR Biosciences) according to manufacturer instructions and quantified by densitometry with Image J software. Cropped images are shown as part of main figures; full-length membranes are shown as part of supplementary information (Figure S5).

### Clonogenic efficiency

Cells (500/well) were seeded on 6-well plates. After attachment, they were cultured in the presence of Met (2.5 mM) and/or Prop (2.5 µM) for 7–10 days as described before^23^. After fixing the cells with formalin [4% PBS-buffered p-formaldehyde (Anedra)], colonies were stained with methylene blue (1%, Sigma) to allow quantification. Photos of the clones were taken at the end of the experiment and their size was estimated by measuring colonies diameters with the Image J software.

### Generation of 5-FU resistant HCT116 cells

To generate 5-FU resistance on HCT116 cells (HCT5FUR) we proceed as described before^74,75^. Briefly, cells were exposed to increasing doses of 5-FU. During the first three weeks cells were treated with 1 μM 5-FU (estimated as half the IC50 value for 5-FU on HCT116 cells). Cells were allowed to recover and replated until they reached 70% confluence. Then, cells were exposed to 2.5 μM 5-FU for three weeks, allowed to recover and replated. Next, the cells were exposed to 5 μM 5-FU for three weeks (twice the IC50 value). Finally, during two weeks cells were exposed to 4 μM 5-FU (twice the IC50 value). Surviving cells were replated and maintained in complete media with 5 μM 5-FU.

### Retroviral transduction

Genetic manipulation of 4T1 cells to stably expressing GFP was performed by transduction with a retrovirus-based plasmid as previously described^76^. Briefly, retroviral particles were generated by co-transfection of 293 GP packaging cells with the packaging plasmid pMD2ENV and pLPC-GFP vector^77^ using the standard calcium-phosphate method. Retroviral particles were harvested 48 h after transfection in the culture media and used to infect 4T1 cells. Upon infection, cells were allowed to recover for 24 h in fresh media and then puromycin (2 μg/ml) was added to allow selection. Infected cells were enriched by puromycin selection for at least one week. GFP expression was confirmed by western blot and fluorescence microscopy.

### Animal studies

BALB/c and athymic nude mice were obtained from the School of Veterinary Sciences at the National University of La Plata and treated in accordance with the Canadian Council on Animal Care and ARRIVE guidelines. Animals were fed with commercial chow and water ad libitum, and maintained in a 12 h light/dark cycle at the CIPReB facilities (Centro de Investigación y Producción de Reactivos Biológicos, Medicine School, National University of Rosario). For all experiments, animals (N = 5–8/group) were distributed and treated as follows: Control: regular drinking water; Met: metformin in drinking water (400 mg/kg BW/day); Prop: propranolol in drinking water (7 mg/kg BW/day); M + P: Met + Prop treatments combined.

### Chemically induced carcinogenic model

8 weeks-old BALB/c female mice were intraperitoneally injected with azoxymethane (Santa Cruz Biotechnology) and then treated with dextran sulfate sodium salt (Santa Cruz Biotechnology) in the drinking water as described by Neufert^78^. Two months after the last round of dextran sulfate, animals were distributed in the groups and treated for four weeks. Then, the animals were sacrificed, intestines were removed and the number of tumors in both groups quantified. To highlight tumor formations, colon was stained with 0.5% methylene blue and visualized under a magnifying glass. Tumors were collected for histology.

### Xenograft and isograft models

2 × 10^6^ HCT116 or 5 × 10^5^ CT26 viable cells were resuspended in PBS (100 μl) and injected subcutaneously in a group of 8-week-old female nude mice.

5 × 10^3^ viable 4T1 cells were resuspended in PBS (100 μl) and injected orthotopically into the fourth right mammary gland of 8-week-old BALB/c female mice. In all the cases, ten days later, mice were distributed in groups and treated as described before^23^. Primary tumor growth was analyzed by measuring tumor length (a) and width (b) with a caliper, and by calculating tumor volume (V) with the formula V = 0.4ab^2^. Fitting to exponential growth and tumor volume doubling times were calculated with the GraphPad Prism 7 software. Body weight of the mice was also recorded. After sacrifice, tumors, spleens, lymph nodes and lungs were extracted, fixed in 4% formaldehyde for 24 h and embedded in paraffin for hematoxylin/eosin staining.

### Histological techniques

To evaluate differences in tumor cell proliferation, 6-µm-thick sections were blocked with horse serum, endogenous peroxidase was blocked with hydrogen peroxide and the slides were incubated overnight with rabbit monoclonal antibody to Ki67 (Cell Signaling, 1:400 dilution) or mouse monoclonal anti-E-cadherin (Cell Signaling, 1:100 dilution). Tissue slides were rinsed with TBS-Tween and incubated for 30 min with secondary antibody (Vectastain Elite ABC kit; Vector Laboratories, USA) then developed with diaminobenzidine (BD Pharmigen) and counterstained with hematoxylin. The samples were observed under an optical microscope with a 100 × immersion objective. Same number of pictures was taken per piece of tissue, and Ki67 positive cells were quantified in each field (5 independent fields were quantified per sample, n = 3 per group).

### Metastasis steps characterization

For intravasation analysis, 4T1 cells were injected orthotopically as described before. Animals were distributed in two groups and treated in drinking water (control and M + P). When tumors were exponentially growing (day 23), blood was collected from the maxillary vein and cells were stained with EpCAM (BD Pharmigen, 1:200 dilution), CD34-Biot (eBioscience, 1:200 dilution) Streptavidin-APC (BD Pharmigen, 1:200 dilution) and CD45-APC (BD Pharmigen, 1:200 dilution). The percentage of EpCAM^+^CD34^−^CD45^−^ cells was analyzed by flow cytometry. In parallel, a similar approach was followed injecting 4T1-GFP cells and detecting GFP positive cells by flow cytometry.

To characterize survival and extravasation of circulating breast cancer cells, 4T1 cells were labeled with 5 µM of green cell tracker (CellTracker Green CMFDA, Invitrogen) for 30 min and injected intravenously (i.v) into BALB/c mice (5 × 10^4^/100 μl). A group of these cells received in vitro treatment (5 mM Met + 5 μM Prop) before injection. Mice were separated as follows: (G1) untreated mice injected with control cells; (G2) untreated mice injected with in vitro pretreated cells; (G3) mice receiving M + P injected with control cells. After 48 h, mice were injected i.v with a rhodamine-conjugated lectin I (Vector Laboratories) to stain lung capillaries and were sacrificed one hour later. Blood was collected and subsequently analyzed by flow cytometry to determine positive circulating green cells. At the same time, lungs were excised, fixed and paraffin embedded. 30-μm-thick sections were mounted and examined by confocal microscope to visualize tumor green cells.

### Experimental model to test adjuvant treatment

4T1 and M-406-derived cells were injected orthotopically as described before^23^. Mice were anesthetized by intraperitoneal injection of ketamine/xylazine (100 mg/kg and 10 mg/kg, respectively). Tumors were surgically removed when they reached 100 mm^3^ and mice were distributed in two groups and treated as indicated. After 6 weeks, animals were euthanized, the chest cavity was exposed through a midline chest incision, the trachea cannulated with a 20-gauge needle, and lungs slowly inflated using 1 ml of India ink (Pelikan, 1:16 dilution in PBS). Lungs were then extracted, immersed in Fekete’s solution (100 ml 70% ethanol (Cicarelli), 10 ml 4% formaldehyde (Anedra), and 5 ml 100% glacial acetic acid (Cicarelli)) to destain and metastatic nodules counted *de visu*.

### Statistics

Unless otherwise indicated, data are expressed as the mean ± s.e.m. and are representative of at least three experiments. Parametric distributions were analyzed using Student’s *t* test (when comparing two experimental groups) or ANOVA followed by either Dunnett’s (when comparing more than two experimental groups with a single control group) or Tukey’s HSD test (when comparing more than two experimental groups with every other group). Nonparametric distributions were analyzed using either Mann–Whitney (for comparisons of two experimental groups) or the Kruskal–Wallis followed by Dunn’s (for comparisons of three or more than three experimental groups) tests. The percentage of mice with metastases was analyzed by chi-squared test. In all cases, p values lower than 0.05 were considered statistically significant.

### Ethical approval statement

Authors declare that all experimental protocols involving animals were approved by the “Committee for the Care and Use of Laboratory Animals (CICUAL)” of the Rosario Medical School.

## Supplementary Information

Supplementary Information 1.

Supplementary Information 2.
