# Supplementary material for: Repositioning metformin and propranolol for colorectal and triple negative breast cancers treatment
Source: Sci Rep. 2021 Apr 14;11:8091. doi: 10.1038/s41598-021-87525-z (PMC8047046; doi:10.1038/s41598-021-87525-z)

Repositioning metformin and propranolol for colorectal and triple negative breast cancers treatment

LE Anselmino[1,2], MV Baglioni[2,3], F Malizia[1,2], N Cesatti Laluce[1,2], C Borini Etichetti[2,4], VL Martínez Marignac[2,5], V Rozados[2,3], OG Scharovsky[2,3], J Girardini[1,2], MJ Rico[2,3], M Menacho Márquez[1,2,6*].

Running title: Combination of repositioned drugs prevents colorectal and triple negative breast cancers growth and development

Affiliations

[1] Instituto de Inmunología Clínica y Experimental de Rosario. (IDICER, CONICET-UNR). Facultad de Ciencias Médicas (UNR). Santa Fe 3100, Rosario, Argentina.

[2] CONICET

[3] Instituto de Genética Experimental, Facultad de Ciencias Médicas. Santa Fe 3100, Rosario. Argentina.

[4] Instituto de Fisiología Experimental (IFISE, CONICET-UNR). Facultad de Ciencias Bioquímicas y Farmacéuticas (UNR), Suipacha 570, 2000, Rosario, Argentina.

[5] CICYTTP IBIOGEM, CONICET, Diamante, Argentina.

[6] Centro de Investigación y Producción de Reactivos Biológicos (CIPReB), Facultad de Ciencias Médicas. Suipacha 660, Rosario. Argentina

*To whom correspondence should be addressed:

E-mail: mmenacho@conicet.gov.ar / **Phone:** +54-3413615828 / **Fax:** +54-3414804569

Total number of figures and tables: 5 main figures, 5 supplementary figures and 6 supplementary tables.

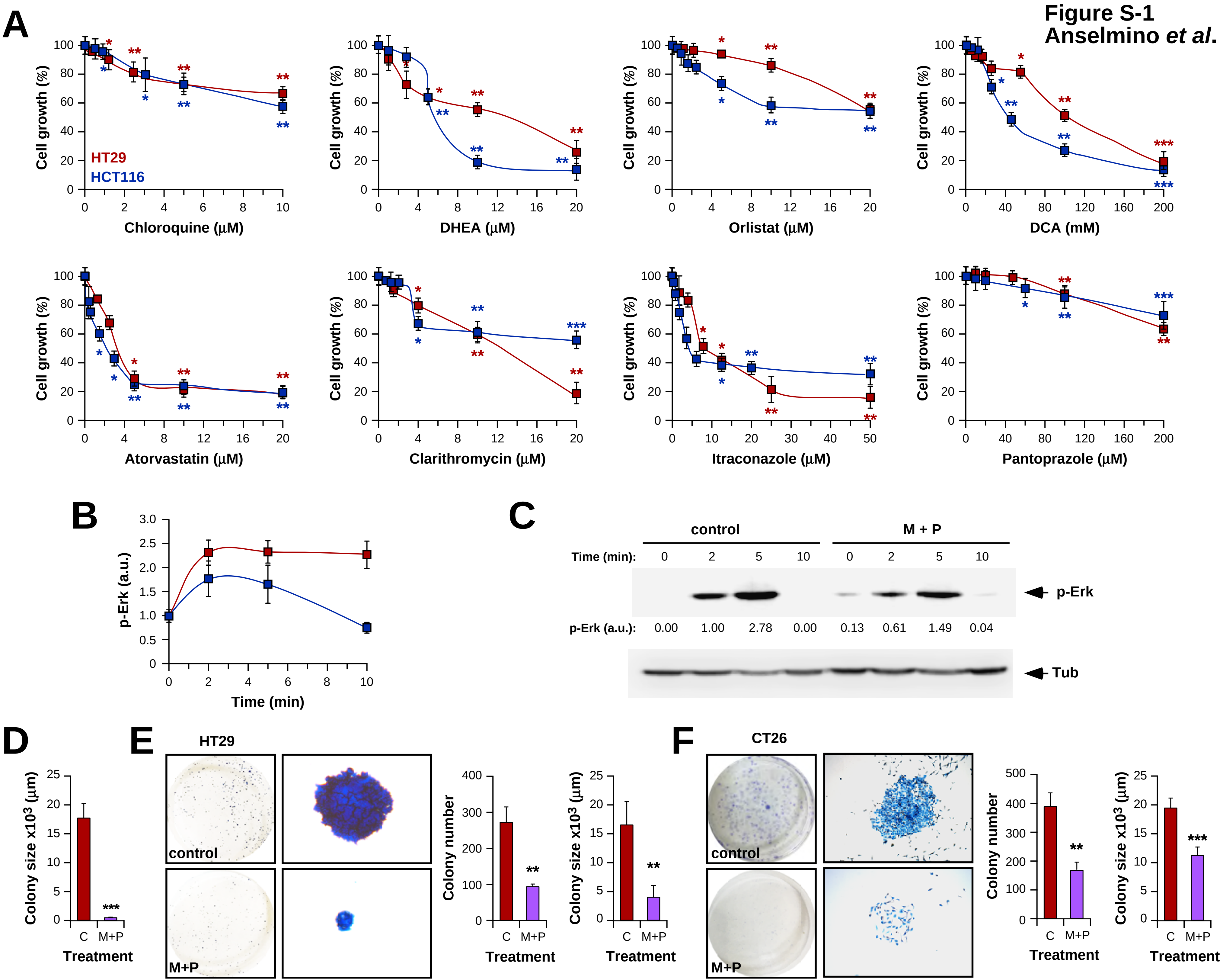
Figure S-1
Anselmino et al.
A
HT29
HCT116
Cell growth (%)
Chloroquine (μM)
DHEA (μM)
Orlistat (μM)
DCA (mM)
Atorvastatin (μM)
Clarithromycin (μM)
Itraconazole (μM)
Pantoprazole (μM)
B
p-Erk (a.u.)
Time (min)
C
control
M + P
Time (min): 0 2 5 10 0 2 5 10
p-Erk
p-Erk (a.u.): 0.00 1.00 2.78 0.00 0.13 0.61 1.49 0.04
Tub
D
Colony size x10³ (μm)
C M+P
Treatment
E
HT29
control
M+P
Colony number
Colony size x10³ (μm)
Treatment
F
CT26
control
M+P
Colony number
Colony size x10³ (μm)
Treatment

Figure S-2
Anselmino *et al.*

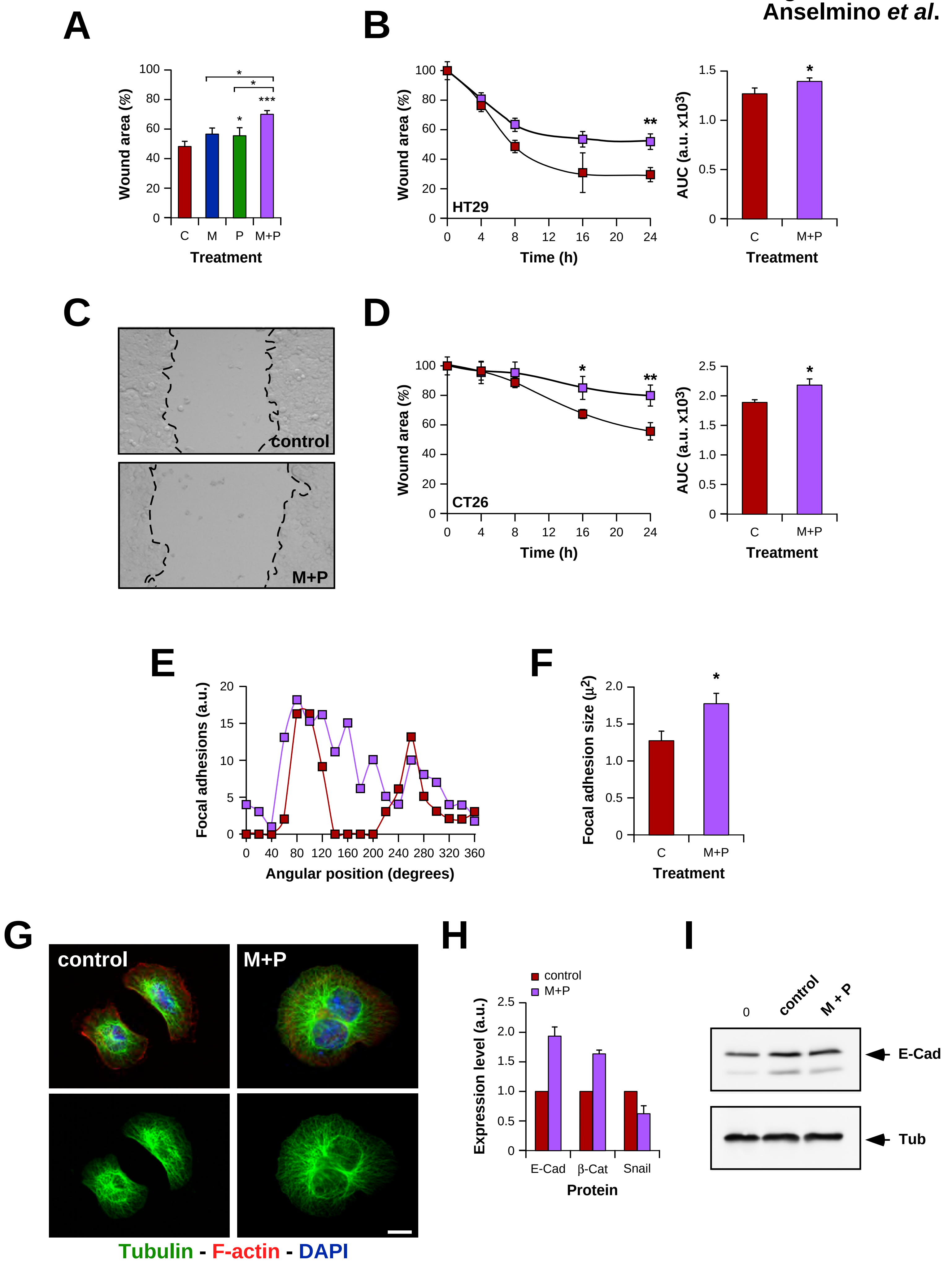

Figure S3
Anselmino *et al*.

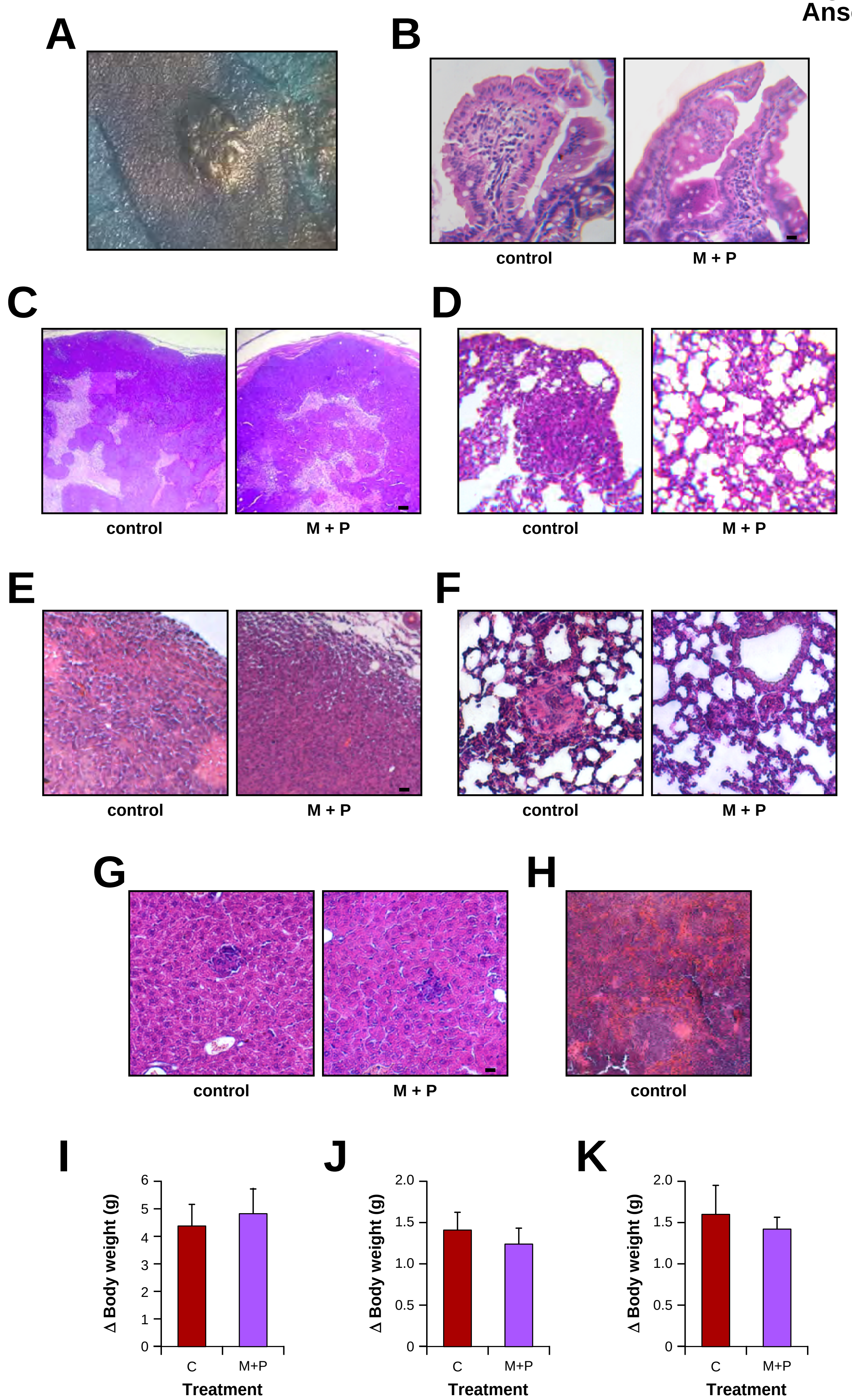

Figure S-4
Anselmino *et al*.

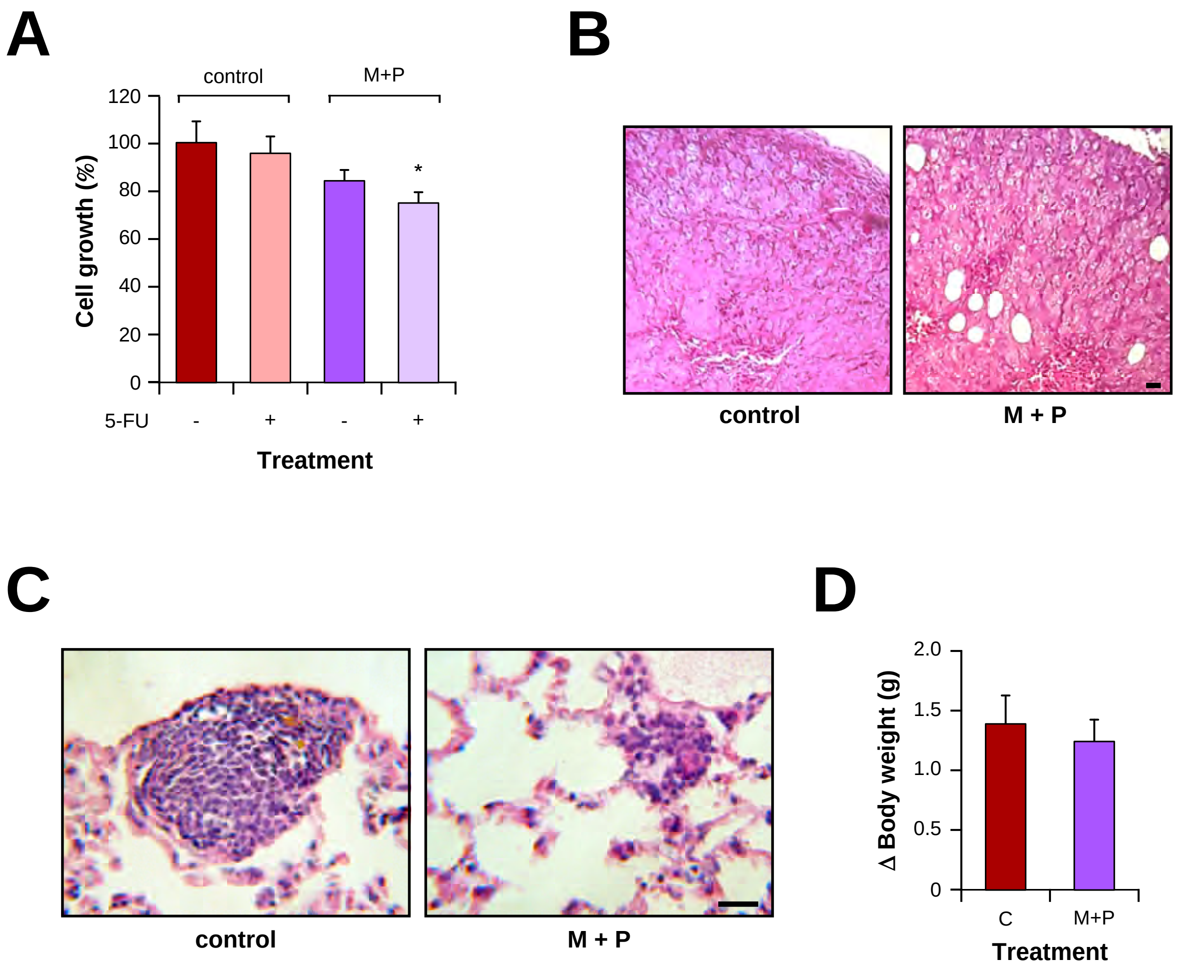

Fig. 1G

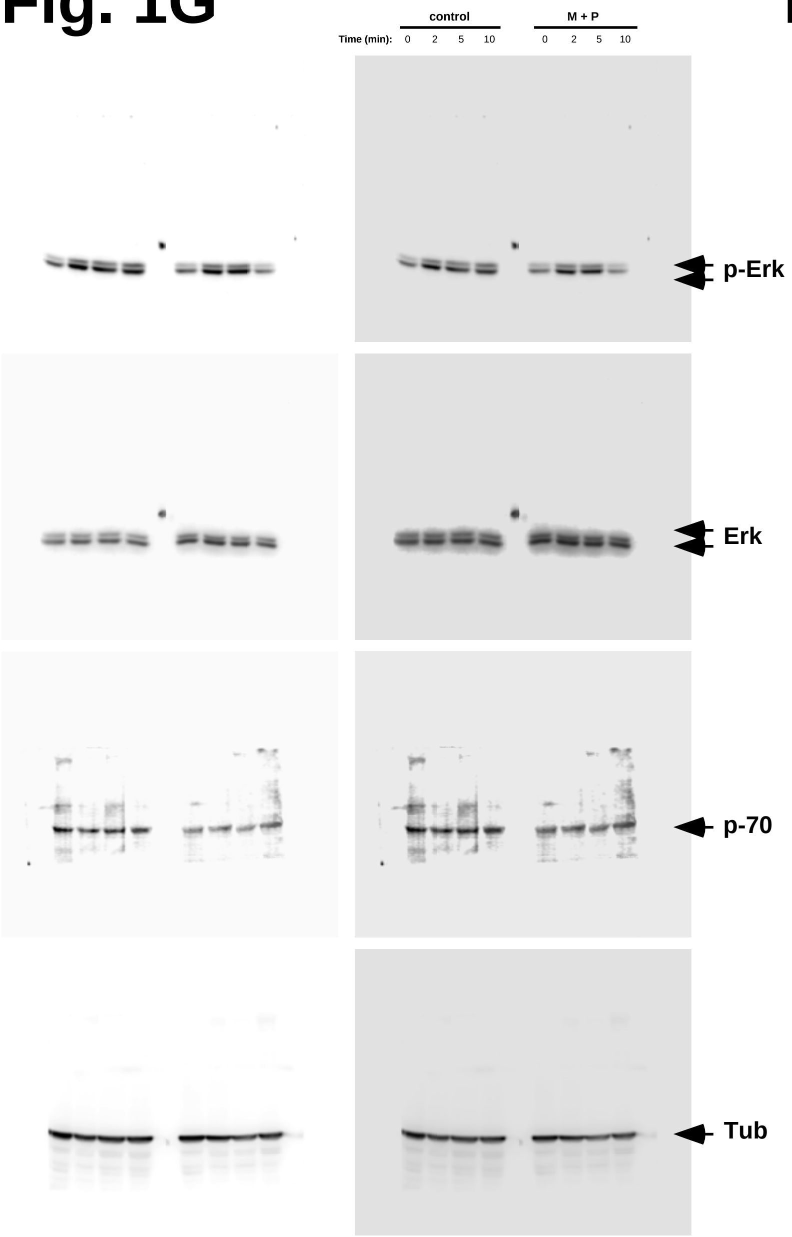

Fig. 2D

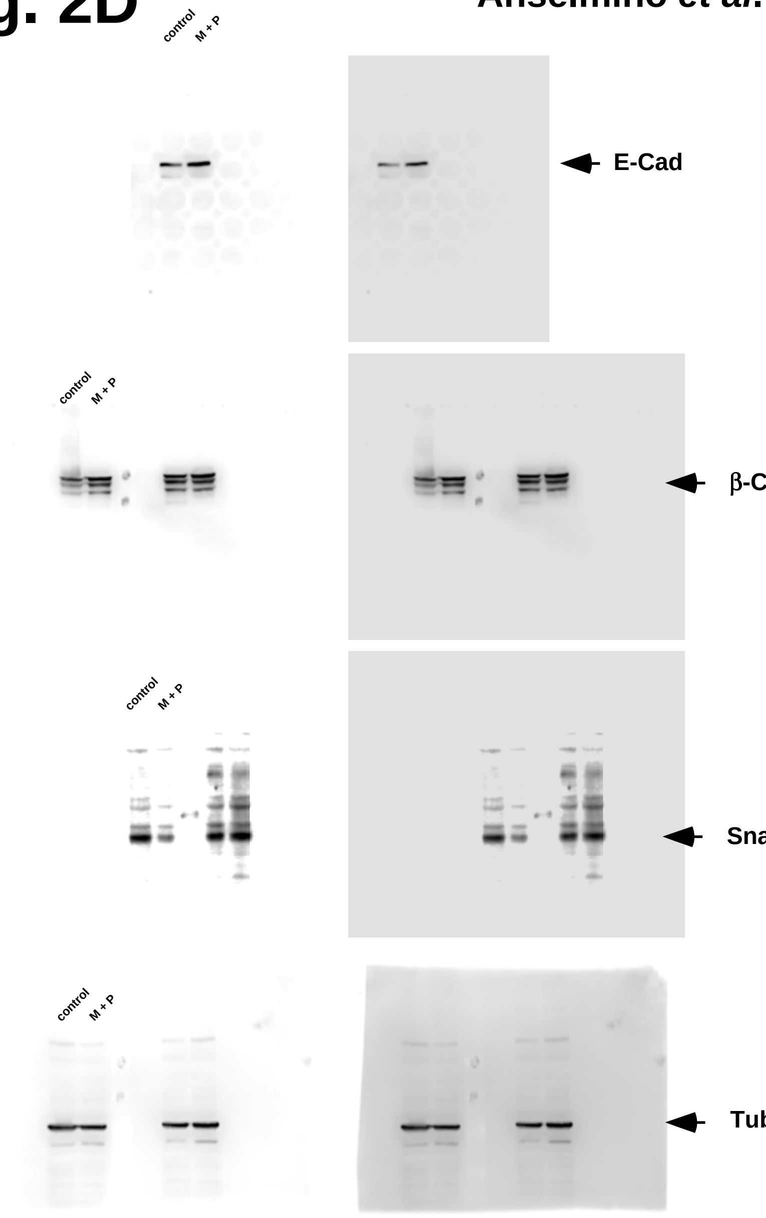

Fig. S1C

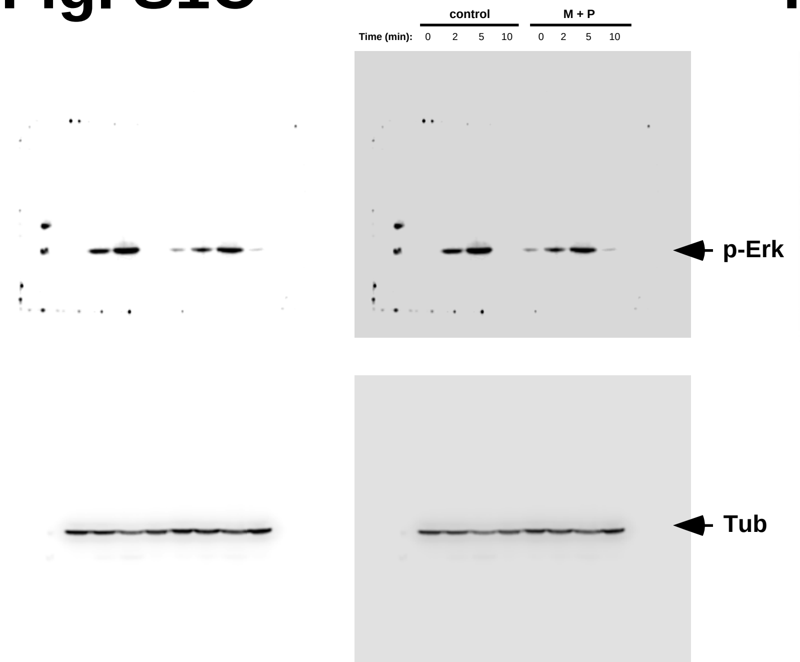

Fig. S2I

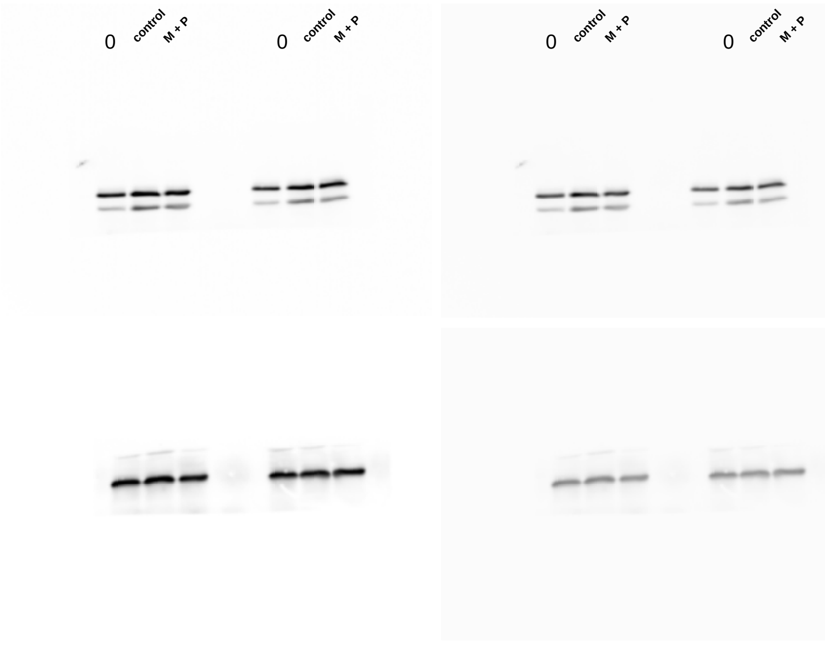

Supplement: Supplementary file 1 — Supplementary Information 1. [file 41598_2021_87525_MOESM1_ESM.pdf]
