# Supplementary material for: Repositioning metformin and propranolol for colorectal and triple negative breast cancers treatment
Source: Sci Rep. 2021 Apr 14;11:8091. doi: 10.1038/s41598-021-87525-z (PMC8047046; doi:10.1038/s41598-021-87525-z)
Supplement: Supplementary file 2 — Supplementary Information 2. [file 41598_2021_87525_MOESM2_ESM.docx]

**FIGURE S1: Effect of a group of repurposing drugs on CRC cells proliferation.** (**A**) CRC cells were cultured in the presence of the indicated doses of the selected drugs during 36 hours. The number of living cells was estimated by tetrazolium salts reduction method (n=3). (**B**) Kinetics of Erk phosphorylation in HCT116 cells after serum stimulus (n=3). (**C**) Western blot analysis showing the phosphorylation and expression status of Erk in serum-stimulated 4T1 cells. Cells were treated for 30 minutes with M+P (M 2.5mM; P 2.5µM) before released in complete media. Tubulin was used as loading control. Quantification of phosphorylation levels is indicated. (**D**) Analysis of colony size of HCT116 cells plated for the clonogenic assay described in Figure 1H. (**E,F**) HT29 (D) and CT26 (E) CRC cells (500 cells/well) were cultured in the presence of M+P during 8 days. Colonies were stained (left panel) in order to allow quantification (middle panel). Colony size was estimated measuring diameters under a microscope (right panel)

**FIGURE S2. M+P affects EMT in CRC cells but not in TNBC cells.** The migratory ability of CRC cells was estimated by classical wound-healing assays. (**A**) Quantification of wound area for HCT116 cells treated with Met (M, 2.5mM), Prop (P, 2.5µM) or their combination (n=3). (**B**) Kinetics of wound closure (left) and quantification of the area under the curve (AUC, right) for HT29 cells treated with M+P. Representative pictures were taken (**C**, time=16h) to allow quantification of healing by using the Image J software. (**D**) Kinetics of wound closure (left) and quantification of the area under the curve (right) for CT26 cells treated as before. (**E,F**) Distribution (E) and size (F) of the focal adhesions observed after FAK and vinculin immunostaining (Figure 2C) using ImageJ software. (**G**) Representative immunofluorescence analysis showing the abundance and subcellular localization of endogenous α-tubulin (green color), rhodamine phalloidin-stained F-actin (red color), and DAPI-stained nuclei (blue color; scale bar=10µm). (**H**) Quantification of E-Cadherin, β-Catenin and Snail levels in HCT116 cells after M+P treatment (24 hours, n=3). (**I**) Representative Western Blot (n=3) showing no significant change in E-Cadherin endogenous expression observed after treating 4T1 cells with M+P. Tubulin was used as a loading control

**FIGURE S3. Histological characterization of colorectal tumors and metastasis.** (**A**) After chemical carcinogenesis tumors were observed under a stereoscopic microscope. (**B**) Haematoxylin eosin staining of tumors derived from control and M+P treated animals after azoxymethane/dextran sulfate carcinogenesis. (**C,D**) Haematoxylin eosin staining of tumors (C) and metastasis (D) derived from control and M+P treated animals after HCT116 xenographic injection. (**E-H**) Haematoxylin eosin staining of tumors (E), lungs (F), liver (G) and intestinal lymph nodes (H) derived from control and M+P treated animals after CT26 subcutaneous injection. No intestinal lymph node was observed for treated animals. (**I-K**) No signs of toxicity associated to M+P treatment as observed by body weight change in carcinogenesis (I) and HCT116 (J) and CT26 (K) xenographic models.

**FIGURE S4. M+P treatment prevents growth and metastasis development of 5-FU resistant cells.** (**A**) Proliferation analysis of 5-FU resistant HCT116 pre-treated during 4 hours with M+P. (**B,C**) Haematoxylin eosin staining of tumors (B) and lungs (C) derived from control and M+P treated animals after 5-FU resistant HCT116 xenographic injection. (**D**) No difference in body weight was observed for animals treated with M+P.

**FIGURE S5. Full-length Blots.** Original figures are indicated in each case.

**SUPPLEMENTARY TABLE I.**

| **Drug** | **HCT116** | **HT29** |
| --- | --- | --- |
| Metformin (mM) | 2.61 +/- 0.24 | 3.11 +/- 0.22 |
| Propranolol | 25.5 +/- 0.28 | 39.04 +/- 0.52 |
| 5-FU | 2.49 +/- 0.16 | 2.54 +/- 0.47 |
| Chloroquine | 2.72 +/- 0.64 | 3.9 +/- 0.50 |
| DHEA | 4.01 +/- 0.18 | 5.74 +/- 0.34 |
| Orlistat | 9.92 +/- 0.62 | 20.01 +/- 0.22 |
| DCA (mM) | 10.98 +/- 0.30 | 50.67 +/- 0.60 |
| Atorvastatin | 1.92 +/- 0.28 | 1.93 +/- 0.10 |
| Clarithromycin | 9.37 +/- 0.20 | 29.37 +/- 0.28 |
| Itraconazole | 1.10 +/- 0.10 | 1.35 +/- 0.14 |
| Pantoprazole (mM) | 0.11 +/- 0.05 | 0.18 +/- 0.08 |

**TABLE SI:** IC50 values of the drugs used in this work. Unless indicated, data are expressed mean +/- standard deviation in μM units.

**SUPPLEMENTARY TABLE II:**

| **Treatment** | **Liver** | **Lungs** |
| --- | --- | --- |
| **control** | 50% (2/4) | 50% (2/4) |
| **M + P** | 0% (0/5) | 0% (0/5) |

**TABLE SII:** Percentage of mice with metastatic nodes detected per mouse by H&E.

**SUPPLEMENTARY TABLE III:**

| **Treatment** | **Liver** | **Lungs** | **Spleen** |
| --- | --- | --- | --- |
| **control** | 100% (4/4) | 75% (3/4) | 100% (4/4) |
| **M + P** | 100% (4/4) | 25% (1/4) | 50% (2/4) |

**TABLE SIII:** Percentage of mice with metastasis in liver and lungs (observed by H&E) and with splenomegaly.

**SUPPLEMENTARY TABLE IV:**

| **Treatment** | **Liver** | **Lungs** |
| --- | --- | --- |
| **control** | 3.25 + 0.48 | 1.50 + 0.64 |
| **M + P** | 2.75 + 0.25 | 0.25 + 0.25 |

**TABLE SIV:** Mean of metastatic nodes detected per mouse by H&E.

**SUPPLEMENTARY TABLE V:**

| **Treatment** | **Mean** | **Lungs** |
| --- | --- | --- |
| **control** | 2 + 0.41 | 100% (4/4) |
| **M + P** | 0.5* + 0.5 | 25% (1/4) |

**TABLE SV:** Mean of metastatic pulmonary nodes per mice and percentages of mice with lung metastasis detected by H&E

**SUPPLEMENTARY TABLE VI:**

| **Name Drug** | **Origin** | **Clinical use** | **Doses** |
| --- | --- | --- | --- |
| Metformin | Sigma-Aldrich | Diabetes | 1-50 mM |
| Propranolol | Sigma-Aldrich | Hypertension | 1-50 μM |
| Chloroquine | Sigma-Aldrich | Malaria | 0.5-10 μM |
| Dehydroepiandrosterone (DHEA) | Parafarm | Sexual hormones precursor | 1-200 μM |
| Tetrahydrolipstatin (Orlistat) | Parafarm | Obesity | 1-200 μM |
| Atorvastatin | Parafarm | High cholesterol treatment | 1-200 μM |
| Dichloroacetic acid (DCA) | Parafarm | Lactic acidosis treatment | 1-200 mM |
| Clarithromycin | Parafarm | Antibiotic | 1-200 μM |
| Itraconazole | Parafarm | Antifungal | 0.1-50 μM |
| Pantoprazole | Parafarm | Gastroesophageal reflux treatment | 5-500 μM |

**TABLE SVI.** Drugs, description and doses used in this work.
